# Research progress of NF-κB signaling pathway and thrombosis

**DOI:** 10.3389/fimmu.2023.1257988

**Published:** 2023-09-29

**Authors:** Zilong Wang, Chucun Fang, Mengting Yao, Dongwen Wu, Maga Chen, Tianting Guo, Jianwen Mo

**Affiliations:** ^1^ The First Clinical College, Gannan Medical University, Ganzhou, Jiangxi, China; ^2^ Department of Orthopedics, Ganzhou City Hospital, Ganzhou, Jiangxi, China; ^3^ Department of Orthopedics, The First Affiliated Hospital of Gannan Medical College, Ganzhou, Jiangxi, China

**Keywords:** thrombosis, NF-κB signal pathway, inflammation, miRNA, TCM, natural compounds, drugs

## Abstract

Venous thromboembolism is a very common and costly health problem. Deep-vein thrombosis (DVT) can cause permanent damage to the venous system and lead to swelling, ulceration, gangrene, and other symptoms in the affected limb. In addition, more than half of the embolus of pulmonary embolism comes from venous thrombosis, which is the most serious cause of death, second only to ischemic heart disease and stroke patients. It can be seen that deep-vein thrombosis has become a serious disease affecting human health. In recent years, with the deepening of research, inflammatory response is considered to be an important pathway to trigger venous thromboembolism, in which the transcription factor NF-κB is the central medium of inflammation, and the NF-κB signaling pathway can regulate the pro-inflammatory and coagulation response. Thus, to explore the mechanism and make use of it may provide new solutions for the prevention and treatment of thrombosis.

## 1 Introduction

Deep-vein thrombosis (DVT) is a serious disease threatening human life. The incidence of DVT is 10-40% after general surgery and 40-60% after major orthopedic surgery, and in the absence of preventive measures, DVT can lead to further diseases such as pulmonary hypertension, recurrent thrombosis, post-thrombotic syndrome, and even fatal pulmonary embolism (1, 2). Therefore, patients with acute DVT face a high risk of death. The treatment of DVT in modern medicine can be roughly divided into anticoagulation therapy and thrombectomy (3). Anticoagulation therapy and surgery have made great progress in the treatment of DVT and are widely used. If more methods can continue to be found to treat thrombosis, then the recovery probability of related patients will be increased. Therefore, research on the prevention and treatment of thrombosis has never stopped.

Blood flow retardation, blood hypercoagulability, and endothelial cell injury are the three major factors of venous thrombosis (4, 5). They play different roles in the mechanism of venous thrombosis but are related to each other. Vascular endothelial cells divide blood from subendothelial tissue, store and secrete factors that affect platelet function, prevent platelet adhesion, and allow blood to flow normally. However, when the endothelium is disturbed by physical or chemical factors, the endothelial cells will undergo programmed biochemical changes, transform into the front surface of the thrombus, express TF, and accelerate the activation of factor X and factor IX, thereby activating the coagulation system (6). Changes in hemodynamics promote changes in the state of the vascular endothelium, and the flow of blood through the vasculature generates wall shear stress, resulting in structural and functional changes in the vessel wall (7). Shear stress also strongly affects endothelial cell gene expression (8, 9). There are “shear-stress response elements” in the promoters of related genes (10, 11), various “mechanical transducers” and downstream signal pathways, which associate external mechanical stimuli with intracellular and nuclear events (12–14). The hypercoagulable state is one of the important factors of venous thrombosis (15). When too many clotting proteins are produced in the blood, abnormal clotting proteins are produced to resist decomposition, and too few proteins that prevent thrombosis are produced, which will cause the blood to become hypercoagulable (16). The combination of the hypercoagulable state and acquired risk factors (surgery, braking, or hormone therapy) increases the risk of thrombosis (17, 18). Proper thrombus prevention can prevent the risk of thrombosis from exceeding this critical threshold, but thrombosis occurs when internal and external forces exceed the critical threshold (19).

In recent years, studies have found that inflammation is closely related to the formation and development of deep venous thrombosis. Inflammation mediates vascular endothelial cell injury (20, 21), releases vascular cell adhesion molecules (VCAM) and intercellular adhesion molecules (ICAM), stagnates blood flow, and accelerates venous thrombosis (22–25). The formation of blood clots exacerbates the inflammatory response, and the two affect each other. Therefore, study of the occurrence of venous thrombosis and the discovery of related proteins regulating inflammatory factors are of great significance for delaying and alleviating the formation of venous thrombosis and judging the prognosis.

## 2 Overview of the NF-κB signaling pathway

The NF-κB family consists of a group of structurally related and evolutionarily conserved transcription factors that play a key role in inflammatory response, immune function, cell survival, and prevention of apoptosis (26). There are currently five members of the mammalian NF-κB family, known as RelA (also known as p65), RelB, c-Rel, NF-κB1 (p50 and its precursor p105), and NF-κB2 (p52 and its precursor p100) (27).

Despite the expanding complexity of NF-κB signaling, the two most recognized pathways in mammalian cells are the so-called classical and atypical pathways (28–30), both of which are important in inflammatory response and immune regulation, despite their differences in signaling composition and biological function. The classical NF-κB pathway is induced by pro-inflammatory cytokines and depends on the induced degradation of IκB, specifically Iκbα, to activate the NF-κB1 p50, RelA, or c-Rel complex (28). The non-classical NF-κB pathway is triggered by certain members of the TNF family of cytokines rather than by TNF-A itself and depends on the induction process of p100 rather than the degradation of IκBα, leading to the activation of the NF-κB2 P52 or RelB complex (31–33). Activated NF-κB is transferred from the cytoplasm to the nucleus, where it causes the expression of target genes associated with inflammation. The NF-κB family has been shown to activate more than 500 inflammation-related genes (34, 35) and can initiate the expression of cytokines necessary for inflammation. Some of these cytokines, such as IL-1 and TNF-α, activate NF-κB itself, leading to the formation of a positive feedback loop that has the potential to produce chronic and excessive inflammation when NF-κB becomes abnormally or persistently active.

## 3 Relationship between NF-κB, inflammation, and thrombosis

### 3.1 The inflammatory response promotes thrombosis

In general, DVT can be caused by a variety of risk factors, including genetics, dietary habits, obesity, aging, trauma, and cancer (2). In recent years, with the deepening of research, inflammation is considered to be an important way for various risk factors to trigger the formation of VTE (36–39). In our recent case, the novel coronavirus infection (COVID-19) is caused by the novel coronavirus SARS-CoV-2, which is characterized by an excessive inflammatory response. It has been reported that about half of the hospitalized patients with COVID-19 have serious symptoms, such as deep-vein thrombosis and coagulation dysfunction in the lower extremities, and some patients may die (40–44). It has also been reported that the injection of the COVID-19 vaccine can cause an immune inflammatory response, thus promoting thrombosis and thrombocytopenia (45–50). When studying the changes of inflammatory factors in the plasma of DVT patients, it was found that the expression level of IL-17A was up-regulated, and the level of platelet aggregation was increased, which promoted platelet activation and aggregation, thus playing a role in promoting the formation of DVT (51). In addition, pro-inflammatory factors represented by interleukin-1 (IL-1), IL-6, IL-1β, IL-18, cox-2, TNF-α (52–55), and other inflammatory factors can induce inflammatory response, accelerate tissue injury, and stimulate the release of inflammatory mediators, leading to vascular endothelial injury and apoptosis (20, 21). This indicates that inflammation has become a factor that cannot be ignored in the mechanism of thrombosis.

### 3.2 NF-κB induces an inflammatory response

Transcription factor NF-κB is the central mediator of inflammatory response, mainly in the form of p65 and p50 binding and in the form of dimer; when stimulated, NF-κB p65/p50 dissociates with IκBα and enters the nucleus to activate the corresponding gene transcription (56). The NF-κB signaling pathway is the central link of various inflammatory responses, which can up-regulate the expression of pro-inflammatory factors in the activated state, and inflammatory response can release inflammatory factor IL-1β through the NF-κB pathway, resulting in increased expression of monocyte chemotactic protein-1 (MCP-1) and activation of endothelial cells (57, 58). Releasing intercellular adhesion molecule-1 (ICAM-1), platelet endothelial cell adhesion molecule-1 (PECAM-1), and vascular cell adhesion molecule-1 (VCAM-1), etc. (59, 60), further activates NF-κB, amplifies inflammatory response, and releases more inflammatory factors. Increased platelet reactivity, activation of the plasma coagulation cascade, and impaired function of physiological anticoagulants result in the hypercoagulability of blood (61, 62).

### 3.3 NF-κB is involved in thrombosis

NF-κB signaling plays an important role in the vascular system and in the cell types involved in thromboinflammatory processes. By mediating the interaction between endothelial cells, platelets, and inflammatory response, NF-κB disrupts the coagulation–fibrinolysis balance and induces thrombosis (63).

#### 3.3.1 Platelets

Platelets are not only involved in primary hemostasis but also in the formation of thrombus induced by inflammation. Platelet activators include not only thrombin and ADP but also molecules involved in inflammation (64). Platelets as non-nucleated cells also contain members of the NF-κB family and their corresponding signaling molecules, which are involved in platelet activation and secondary feedback loops (56). Activated platelets express or secrete pro-inflammatory and pro-coagulant substances on their surfaces, such as adhesion molecules, growth factors, cytokines, and the fibrinolytic inhibitor PAI-1, inducing surface aggregation of coagulation factors (65).

#### 3.3.2 Endothelial cell

The injury of the vein wall or vein endothelial cells caused by various factors is one of the factors that cause DVT. Studies have confirmed that NF-κB signaling molecules exist in endothelial cells (63). When injured, endothelial cells are activated, which can inhibit the expression of thrombomodulin (TM) and activate the expression of endothelial tissue factor (TF), resulting in the activation of adhesion molecules such as P-selectin and clotting factor vWF (66), and the endothelial cells change from anticoagulant, anti-inflammatory, and vasodilator functions to proinflammatory and pre-thrombotic states.

#### 3.3.3 NETs

**Figure 1:**
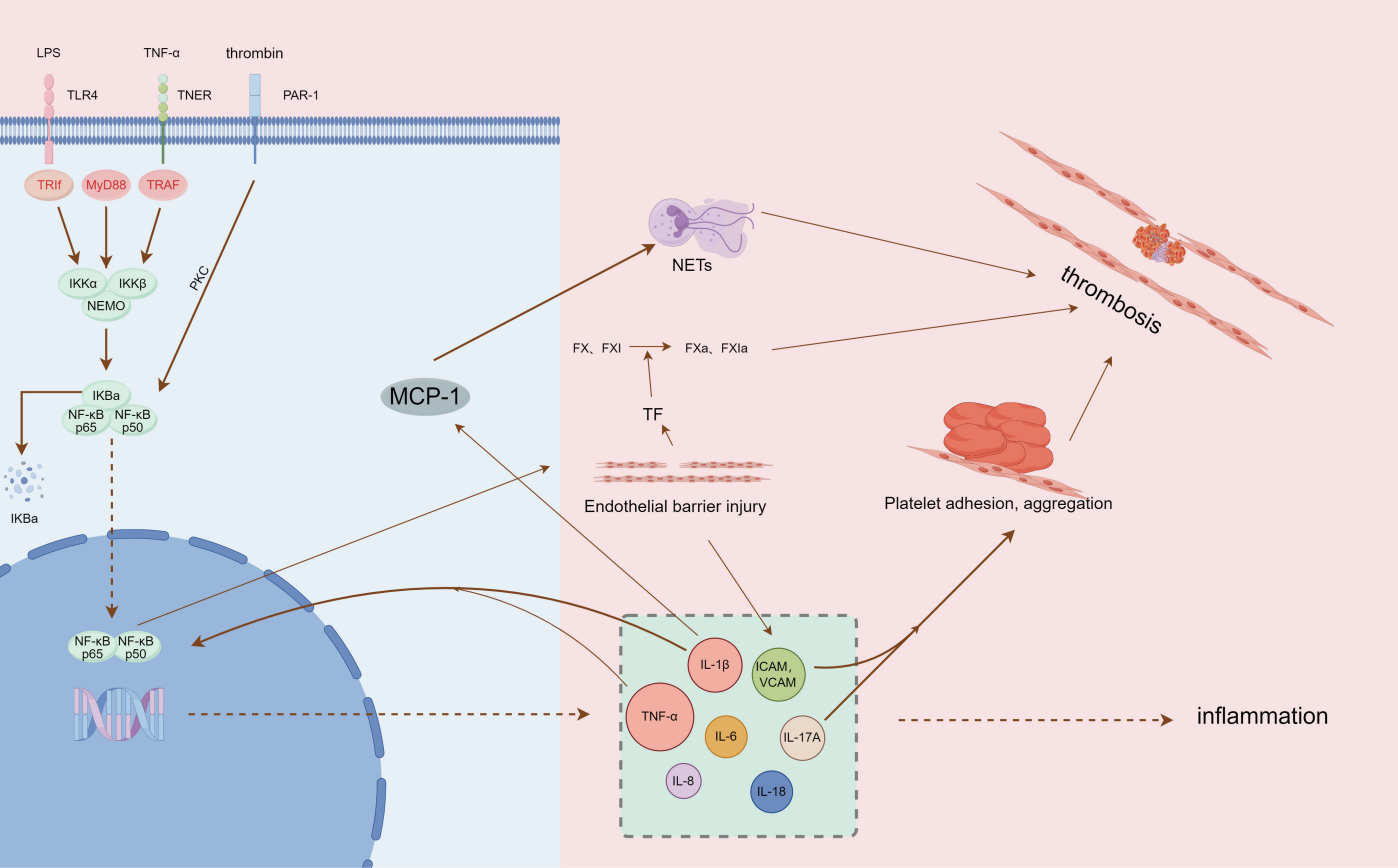
Summary of NF-κB pathway mediating inflammation and thrombosis (by Figdraw, authorization ID: PIWYI6b557).

Inflammatory cells represented by neutrophils and monocytes were able to rapidly aggregate and adhere to the venous endothelium (67). Among them, neutrophils can activate coagulation factors XII, initiate endogenous coagulation, and also form neutrophil extracellular traps (NETs) after apoptosis. NETs, as part of the body’s innate immunity, are an extracellular network of fibers made up of disaggregated chromatin (DNA fibers and histones) released by neutrophils and more than 30 granule proteins with antimicrobial properties (68, 69), providing a supporting basis for thrombosis through their fiber network structure (70). They interact with platelets to further stimulate platelet aggregation, activate thrombin, and accelerate the DVT process (71, 72) (**Figure 1**).

## 4 Interference with NF-κB signaling pathway and deep-vein thrombosis

The NF-κB signaling pathway is closely related to thrombosis. Activation of the NF-κB signaling pathway can significantly increase the levels of thromboxane B2 (TXB2), interleukin6 (IL-6), tumor necrosis factor-α (TNF-α), and PAI and significantly decrease the levels of 6-keto-PGF1α and t-PA, exacerbating inflammation and thrombosis (73). Therefore, we may think that thrombosis can be prevented and that it can be reduced by inhibiting the NF-κB signaling pathway.

### 4.1 microRNA regulates NF-κB signaling pathway to interfere with deep-vein thrombosis

MiRNAs are key regulators of many biological processes (cell differentiation, proliferation, apoptosis, and metabolism), and abnormal expression of miRNAs is known to be associated with a variety of human diseases. Hugo’s (2016) study demonstrated the abnormal expression of miRNA in venous thrombosis and suggested that miRNA may be related to the molecular mechanism of DVT. There is evidence that miRNA plays an important role in hemostasis (74), and some foreign scholars (75) have confirmed that miR-181a-5p can inhibit the expression of F11 mRNA and coagulation factor XI. Some members of the miRNA family (miR-126 and miR-145) can promote the dissolution and recanalization of thrombus (76, 77), suggesting that miRNA may be involved in the formation of DVT.

#### 4.1.1 miRNA-181b

DVT group and miR-181b overexpression and inhibition of rat models were constructed by producing a deep-vein thrombosis model and injecting normal saline, miR-181b mimics, and inhibitors into the tail vein. The changes of NF-κB (P65) in the venous endothelium of rats in each group were analyzed, and the expression level of NF-κB (P65) in the venous endothelium of rats in the Normal group was used as a reference. The results showed that the expression of NF-κB (P65) in the venous endothelium of rats in the 181b-i group was the highest, followed by the DVT group, and the 181b-m group was lower than the DVT group but higher than the Normal group; moreover, the difference was statistically significant (P<0.05). These results indicate that the expression of NF-κB (P65) is increased in the rat vena cava DVT model, and miR-181b can inhibit the expression of NF-κB (P65). By comparing the length and wet weight of the thrombus in each model group, no thrombus formation was observed in the Normal group. Overexpression of miR-181b can shorten the length of the thrombus and lighten the wet weight after thrombus formation in rats; inhibition of miR-181b expression can lengthen the length of thrombus and increase the wet weight after thrombus formation. These results suggest that miR-181b can reduce the formation of DVT by inhibiting the NF-κB signaling pathway to a certain extent (78).

#### 4.1.2 miRNA-150

Up-regulating the expression of miR-150 inhibited thrombosis in DVT rats. The effect of miR-150 on inflammation was studied *in vivo* and *in vitro*. The rats were injected with LV-NC, and it was found that the administration of LV-miR-150 inhibited the platelet aggregation inhibition rate and TXB2 content in the rat model and significantly inhibited thrombosis. Transfected into *in vivo* skin cells, PAI-1, TNF-α, IL-6, and IL-8 levels were significantly reduced, indicating that miR-150 alleviated inflammation and inhibited apoptosis of vascular endothelial cells. The expression level of NF-κB p50 in vascular endothelial cells transfected with miR-150 mimics was significantly decreased, while the expression level of NF-κB p50 was significantly increased by miR-150 inhibitors. These results suggest that miR-150 may have a negative regulatory effect on NF-κB p50. Therefore, overexpression of miR-150 can be used as a potential therapeutic target for future DVT (79).

#### 4.1.3 miRNA-141

This study found that by overexpression of miRNA-141, the expression of TLR4 and its signaling pathway-related proteins NF-κB, Rac1, and IL-1β in vascular tissues of thrombotic rats was significantly down-regulated, and by restoring the expression of TLR4 and NF-κB, the expression of Rac1 and IL-1β was restored at the same time, and multiple related indexes of miRNA-141 on thrombus were significantly reversed (80). Activated TLR4 induces late activation of NF-κB, which significantly increases TNF-α expression and causes widespread inflammation (53). Furthermore, TNF-α stimulates the TNF receptor (TNFR) and induces phosphorylation of IκB kinase (IKK), which in turn enhances NF-κB activity (81). NF-κB is the end point of the TLR4/NF-κB pathway and the regulatory hub of an inflammatory response, and its activation can enhance the inflammatory response and promote the formation of a thrombus. This may be a key mechanism by which miRNA-141 inhibits thrombus formation by regulating the TLR4/NF-κB signaling pathway (**Table 1**).

**Table 1:** The effect of miRNA on NF-κB-mediated inflammatory response.

MiRNA	Anti-inflammatory effect	Action target	reference
miRNA-181b	e-selectin、VCAM-1、ICAM-1	NF-κB p65	(78)
	Thrombus length and wet weight		
miRNA-150	PAI-1、TNFα、IL-8、IL-6	NF-κB p50	(79)
	Reduce the degree of vascular obstruction; Endothelial cell proliferation was enhanced		
miRNA-141	Rac1、IL-1β	TLR4、NF-κB	(80)
	Inhibit thrombosis and platelet aggregation		

### 4.2 TCM preparations alleviate thrombosis by modulating NF-κB signaling

#### 4.2.1 *Qihong Tongluo* prescription

*Qihong Tongluo* prescription is mainly composed of Astragalus and safflower. Astragalus has a variety of biological functions, including potent immunomodulatory, antioxidant, anti-inflammatory, and antitumor activities (82). Isoflavones, saponins, and polysaccharides are three types of beneficial compounds for their pharmacological activity and therapeutic efficacy (82–84). Astragalus polysaccharides decreased the expression of IL-1β, IL-6, TNF-α, and INF-γ by regulating the toll-like receptor 4 (TLR4)/NF-κB signaling pathway (85, 86). In addition, safflower can inhibit platelet activation, adhesion, and aggregation (87, 88). In summary, we believe that Qihong Tongluo prescription’s inhibition of thrombosis is the result of the joint action of its active components.

#### 4.2.2 Gegen Qinlian pills

The high incidence of thrombotic events is one of the clinical features of coronavirus disease (COVID-19) due to the high inflammatory response caused by the virus. Gegen Qinlian pill (GQP) is a traditional Chinese medicine, which can inhibit toll-like receptor 4 (TLR4)/nuclear factor κB (NF-κB) signaling (89–91), has good anti-inflammatory activity, has a good effect on the treatment of COVID-19, and has shown anti-thrombotic potential. In our study, GQP treatment significantly reduced the expression of TNF-α, NLRP3, and NF-κB, reduced lung, liver, and tail thrombosis, and increased tail blood flow in mice (92). This at least partially supports the hypothesis that GQP can inhibit inflammation-induced thrombosis by inhibiting NF-κB/NLRP3 signaling.

#### 4.2.3 Huanglianjiedu Decoction

HLJJD is a famous prescription in China, and its main compounds have been studied as baicalin and berberine (93). Baicalin, a flavonoid compound extracted from the root of Scutellaria baicalensis, has significant anti-inflammatory and antibacterial effects, scavenging oxygen free radicals and anti-allergic reactions (94, 95). Baicalin can inhibit the NF-κB signaling pathway, reduce the expression level of the p-NF-κB p65 protein, and reduce inflammatory response (96, 97). Berberine is an isoquinoline alkaloid isolated from Coptis chinensis, a Chinese medicinal plant, and has significant anti-inflammatory effects (98, 99). For the infection-induced tissue injury model, the gene and protein expression levels of TNF-α, TLRs, and NF-κB p65 were significantly reduced in the berberine treatment group (100–102). TLR4 is a pattern recognition receptor, and activated TLR4 induces late activation of NF-κB, which significantly increases TNF-α expression and causes widespread inflammation (53). TNF-α stimulates the phosphorylation of IκB kinase (IKK), which in turn enhances the activity of NF-κB (81). As the endpoint of the TLR4/NF-κB pathway and the regulatory hub of the inflammatory response, the activation of NF-κB can enhance the inflammatory response and promote the formation of thrombosis.

Through intravenous injection of the effective components of Huanglian Jidutang into the thrombus model, the results showed that the contents of IL-1β, IL-6, and TNF-α and the expression levels of TLR4, NF-κB, NLRP3, and Caspase-1 were decreased (103), and they also showed significantly reduced thrombus dry weight, Block platelet aggregation, and adhesion induced by collagen (104). In conclusion, the inhibitory effect of the active components of Huanglian Jiedu Decoction on the NF-κB pathway is the cause of alleviating inflammation and reducing thrombosis.

#### 4.2.4 Liu Shen Wan

LSW is a classic proprietary Chinese medicine with anti-inflammatory and analgesic effects (105). Its main components are cow gallstone, musk secretion, toad secretion, pearl shell, realgar, and borneol (106). In PR8-infected cells, LSW significantly down-regulated the expression levels of IL-1β, TNF-α, IL-6, and IFN-γ. In mice infected with PR8, LSW reduced the secretion of TNF-α, IL-1β, IL-6, and IFN-γ in lung tissue, significantly improving survival. In addition, LSW significantly reduced the expression levels of TLR4, phosphor-NF-κB p65, and phosphor-iκBα (107). It is suggested that LSW exerts anti-inflammatory effects by regulating the TLR4/NF-κB signaling pathway.

#### 4.2.5 Rhein

Rhein is widely found in a variety of Chinese herbs, including Rhubarb palmatum, aloe curacao, cassia stenophyllum, and polygonum multiflorum. It has antioxidant, antiviral, anti-inflammatory, anti-tumor, and immunomodulatory activities (108). In mice infected with PR8, rhein significantly improved survival and reduced the lung index and lung expression levels of IL-1β, IL-6, IL-8, and TNF-α. In addition, rhein significantly reduced the protein levels of TLR2, TLR3, and TLR4 and the phosphorylation of NF-κB p65 in PR8-infected cells. The addition of TLR4 and NF-κB activators can antagonize the inhibitory effect of rhein on viral replication (109), so the anti-inflammatory effect of rhein may be related to inhibiting the activation of the TLRs/NF-κB signaling pathway.

#### 4.2.6 Flavonoids

H. cordatum Thunb. is an important plant medicine with antiviral, antibacterial, anti-inflammatory, and antioxidant activities (110–112). Flavonoids, one of the effective components of this phytomedicine, can reduce lung and intestinal damage in mice, inhibit the over-release of tumor necrosis factor-α, IL-1, IL-8, and MCP-1 in the lung tissue of infected mice, and inhibit the up-regulated expression of TLR and NF-κB p65 proteins (113). HCP may play an anti-inflammatory role by inhibiting the activation of the TLR/NF-κB signaling pathway (**Table 2**).

**Table 2:** The effect of traditional Chinese medicine on NF-κB-mediated inflammatory response.

TCM	Single active component	Anti-inflammatory effect	Molecular target	reference
Qihong Tongluo prescription	Astragalus polysaccharide	IL-1β, IL-6, TNF-α, INF-γ, MCP-1	TLR4, NF-κB p65	(85), (86)
	Safflower flower	Inhibited platelet aggregation		(87), (88)
Gegen Qinlian Pills (GQP)	——	TNF-α, IL-6, IL-1β, IL-4	HMGB1, TLR4, NFκB, NLRP3	(89), (92)
		Reduced lung, liver, and tail thrombus formation in mice; increased tail blood flow. The adhesion of platelet to HUVEC was decreased		
Huanglianjiedu Decoction(HLJJD)	baicalin	IL-1β, TNF-α, PEG2	TLR4,NF-κB p65,CD14,p-IKBα/IKBα	(94), (96)
	berberine	TNF – α,ICAM – 1,MCP – 1,IL-1β,IL-6,NLRP3	AMPK,MyD88,NF-κB-p65,TLR4	(98), (100), (103)
Liu Shen Wan(LSW)	——	TNF-α,IL-1β,IL-6,IFN-γ	TLR4,p-NF-κB p65,NF-κB p65,p -κB α	(107)
		The infiltration of inflammatory cells in the lung was reduced		
——	Rhein	It improved the survival rate of mice and reduced lung inflammation	TLR4,Akt,p38,JNK,MAPK,NF-κB	(109)
——	Flavonoids	MCP-1,IL-8,TNF-α,MDA	TLR3/4/7,NF-κB p65	(113)
Xiao shuan jing mai decoction		The expression of miR-181b in venous endothelium of DVT model rats was significantly up-regulated	miRNA-181	(78)

There are studies that Xiaoshuantongmai decoction can mediate microRNA-181b intervention in deep-vein thrombosis. According to the equivalent dose ratio of kg body weight of human and animal, the drug dose was converted, and based on the deep-vein thrombosis model, normal saline and Xiaoshuantongmai decoction were administered. The results showed that the expression level of miR-181b in the venous endothelium of the Xiaoshuantongmai Tang group was the highest, followed by the blank control group and the sham operation group, and the lowest was in the model control group, with a statistical difference. It was proved that Xiaoshuantongmai decoction can up-regulate the expression of miR-181b in the venous endothelium of DVT model rats (78).

### 4.3 Anticoagulant and antiplatelet agents inhibit thrombosis by regulating the NF-κB signaling pathway

#### 4.3.1 Aspirin and salicylate

Acute pulmonary thromboembolism (APE) is a disorder of pulmonary circulation caused by a blockage of the pulmonary artery. Extensive inflammatory responses have been demonstrated in the lung tissue of APE rats, accompanied by significantly elevated levels of tumor necrosis factor-α, interleukin-1-β, and IL-8 (114, 115). High levels of NF-κB were also observed in rats after APE induction (116, 117). Aspirin and salicylate have been reported to inhibit NF-κB equally (118, 119). Healthy SD rats were randomly divided into a control group, sham operation group, APE model group, and aspirin low-dose, medium-dose, and high-dose groups. After APE induction for 6, 24, and 72 h, rats in the low-, medium-, and high-dose aspirin groups were given daily doses of aspirin at 150, 300, and 600 mg/kg, respectively, for 3 consecutive days. The other groups were given the same amount of normal saline. In the APE model group, thrombus formation, alveolar wall injury, pulmonary hemorrhage, and inflammatory cell infiltration occurred at all time points. After aspirin treatment, pathological changes such as pulmonary hemorrhage and inflammatory cell infiltration were reduced. Compared with the APE model group, the expression of the NF-κB protein measured by Western blotting was significantly decreased in other groups at each time point (P<0.05, P<0.001). The highest expression of the NF-κB protein was observed in the APE model group, and NF-κB protein expression decreased gradually in a dose-dependent manner in rats receiving aspirin (120). In summary, aspirin can significantly inhibit the NF-κB pathway in a dose-dependent manner to reduce inflammation and alleviate lung injury after APE.

#### 4.3.2 Platelet P2Y12 receptor antagonist

Clinically, ticagrelor and clopidogrel (antiplatelet coagulants) are often combined with PCI for acute coronary syndromes (ACS) (121). They cure ACS12 adenosine diphosphate (ADP) receptors by targeting the platelet P2Y to inhibit platelet aggregation and reduce thrombosis (122). In this study, human umbilical vein endothelial cells (HUVECs) were cultured with ticagrelor or clopidogrel and given lipopolysaccharide (LPS) and CD14. Human umbilical vein endothelial cells (HUVECs) were cultured with ticagrelor or clopidogrel and given lipopolysaccharide (LPS) and CD14. Ticagrelor and clopidogrel reduce the expression of TNF-α, IL-1, IL-6, IL-8, and IL-2, inhibit p65 phosphorylation and IKB-α degradation, and significantly reduce the amount of nuclear translocation p65 (123, 124). These findings suggest that ticagrelor and clopidogrel inhibit the production of inflammatory cytokines by inhibiting the NF-KB pathway.

#### 4.3.3 The PAR-1 antagonist

PAR-1 can be activated by thrombin to regulate platelet aggregation and endothelial permeability, so it is clinically used as a target for anti-platelet drugs to prevent thrombosis (125–127). Vorapaxar is a representative drug (128). IR induction was performed in rat lung models by perfusion *in vitro*. Male rats were treated with the specific PAR-1 antagonist vorapaxar or the control agent with 40 min of ischemia and 60 min of reperfusion. *In vitro*, mouse lung epithelial cells (MLE-12) were treated with vorapaxar and subjected to hypoxic reoxidation (HR). We found that vorapaxar reduced the production of thrombin, inflammatory factors, cytokine-induced neutrophil chemokine-1, interleukin-6, and tumor necrosis factor-α, pulmonary edema and neutrophil infiltration, and it alleviated lung cell apoptosis and down-regulated the nuclear factor-κB (NF-κB) pathway. It also blocked HR-induced NF-κB activation and the production of inflammatory chemokines in MLE12 cells. The results suggest that vorapaxar acts by blocking PAR-1 expression and modulating the NF-κB pathway (129) (**Table 3**).

**Table 3:** The effect of common antithrombotic drugs on NF-κB-mediated inflammatory response.

Drugs	Representative drug	Anti-inflammatory effect	Action target	reference
anticoagulant	Aspirin and salicylate	Inhibited inflammation and relieved lung injury after APE	NF-κB	(120)
Platelet P2Y12 receptor antagonist	Ticagrelor and clopidogrel	TNFα,IL-1,IL-8,IL-6,IL-2,IL-7,TNF-α,CRP	NF-κB	(123), (124)
		Alleviated LPS-induced cell viability, cell migration and angiogenesis, cell cycle changes, and apoptosis and reduced myocardial ischemia-reperfusion injury (IRI) in ischemic myocardism		
The PAR-1 antagonist	Vorapaxar	CINC-1,IL-6,TNF-α,PAR-1,MPO	PI3K、NF-κB MAPK IκB-α	(129)
		Improved pulmonary edema, pulmonary histopathological changes.		

## 5 Limitations in inhibiting NF-κB signaling pathways

At present, the targeted inhibition of the NF-κB signaling pathway for the treatment of thrombus has not reached the most perfect degree. On the one hand, these drugs/compounds can inhibit the inflammatory response and inhibit the expression of NF-κB, but there is no clear evidence of a targeted relationship, and studies have shown that combination drugs work better (130) and are a more clinically promising therapeutic strategy.

On the other hand, inhibition of the NF-κB pathway is a double-edged sword due to its broad effects. NF-κB mediates cell survival, cell differentiation, and cell proliferation (131), and inhibition of NF-κB has also been shown to play an important role in cancer treatment (132–135). However, long-term use of NF-κB inhibitors can cause side effects such as immune deficiency (136, 137) and intestinal homeostasis imbalance (138), and NF-κB inhibitors should be treated with for a short period. An ideal NF-κB inhibitor would only target the NF-κB pathway without affecting other signaling pathways. However, NF-κB inhibitors interfere with the NF-κB pathway by interfering with other pathways, such as PI3K/Akt and MAPK signaling (87, 115, 117).

## 6 Conclusion

In conclusion, the NF-κB signaling pathway plays an important role in inflammation and deep-vein thrombosis, and the regulation of the NF-κB pathway may bring new strategies for the treatment of thrombosis. Although the role of NF-κB signaling in venous thrombosis has been extensively studied in recent years, the application of NF-κB inhibitors in the treatment of thrombosis has a long way to go, regulating miRNAs or using drugs to interfere with the NF-κB signaling pathway. It may be a potential therapeutic option to improve thrombosis, but the dose and side effects of medication and whether regulating miRNAs will improve other downstream pathways of NF-κB signaling remain to be explored. Given the relationship between inflammation and blood clots, preventing inflammation is a better way to reduce blood clots. Therefore, in-depth study of the mechanism of the NF-κB signaling pathway inducing inflammation will help to elucidate the pathogenesis of venous thrombosis and will also have a far-reaching impact on the development of safer and more effective drugs and the prevention and treatment of thrombosis.

## Author contributions

ZW: Writing – original draft, Writing – review & editing. CF: Investigation, Writing – review & editing. MY: Conceptualization, Investigation, Writing – review & editing. DW: Supervision, Writing – review & editing. MC: Supervision, Writing – review & editing. TG: Supervision, Writing – review & editing, Investigation. JM: Investigation, Supervision, Validation, Writing – review & editing.
